# Genome sequence and annotation of *Arthrobacter globiformis* phage Ruchi (AS1) isolated from soil in Lumpkin County, Georgia

**DOI:** 10.1128/mra.01224-23

**Published:** 2024-03-11

**Authors:** Brooke Tatum, Payton Murray, Claire Bicknell, Shane A. Webb, Alison Kanak

**Affiliations:** 1 Department of Biology, University of North Georgia, Dahlonega, Georgia, USA; Portland State University, Portland, Oregon, USA

**Keywords:** bacteriophage, genome annotation, arthrobacter phage, AS1

## Abstract

Ruchi*,* a temperate, AS1 subcluster bacteriophage isolated in Lumpkin County, Georgia using host *Arthrobacter globiformis*, possesses a genome of 38,571 bp and 67.7% GC. Annotation of this virus revealed 64 predicted reading frames, no predicted tRNA genes, and a close evolutionary relationship to AS1 phage Basilisk.

## ANNOUNCEMENT

Phage therapy presents an alternative approach to the treatment of multidrug-resistant bacterial infections (1–3). A thorough understanding of phage diversity and evolution is therefore paramount. Here, we contribute to this knowledge with the annotated genome of Ruchi, a temperate AS1 subcluster bacteriophage propagated on *Arthrobacter globiformis*.

**Fig 1:**
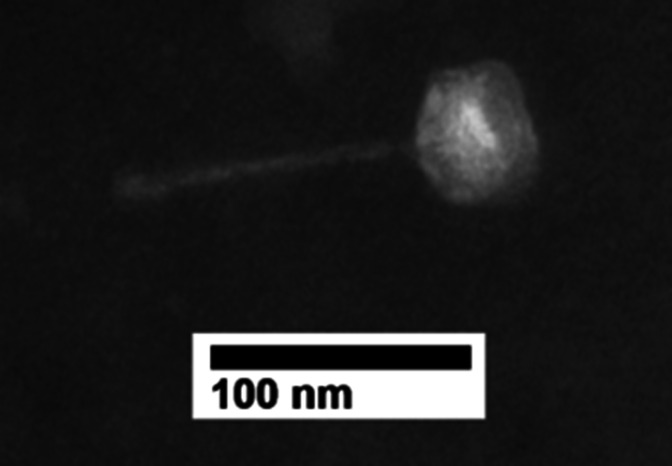
TEM image of phage Ruchi. Lysate was stained using the drop method with phosphotungstic acid. The image was obtained using a JEM-1011 TEM (JOEL, Inc., Tokyo, Japan) at the University of Georgia Electron Microscope Laboratory. Head diameter is ca 44 nm, and tail length is ca 117 nm.

Ruchi was isolated in August 2022 from topsoil from the Pine Valley Recreation Center in Lumpkin County, Georgia (34.51N, 84.06W). Lab methods followed the SEA-PHAGES protocol (4). LB medium was mixed with soil and incubated at 30°C for 1 hour. Supernatant was then sterilized using 0.22 µm filtration. Phage presence was confirmed and purified by standard plaque assay using *A. globiformis* B-2979 and amplified to high titer via flooding of a web plate. A Wizard DNA extraction kit (Promega) was used on 6.7e−9 pfu/mL lysate to obtain 122.2 ng/µL genomic DNA. A NEBNext Ultra II FS kit was used for sequencing library construction. Illumina MiSeq sequencing yielded ~3,914× coverage from 1.1 million 150-bp single-end reads. Genome assembly used Newbler 2.9 (default settings), and the accuracy and completeness of the assembly were evaluated with Consed 29 (5). Ruchi has siphovirus morphology (Fig. 1) and is likely temperate based on plaque morphology and the presence of a tyrosine integrase gene.

DNA Master 5.23.6 (6), Glimmer 3.02 (7), GeneMark 3.26 (8), BLAST (9, 10), HHPred 2.08 (11), Phamerator 505 (12), tRNAscan SE 2.0 (13), Aragorn (14), and DeepTMHMM 1.0.24 (15) were used for genome annotation (all using default parameters). DNA Master provided first-pass analysis of open reading frames (ORFs), gaps, and ribosomal binding sites. Subsequent homology assessment using BLASTp (16) (Genbank nr database), HHPred (default pdb database; UniRef30), and Phamerator refined the annotation. Gaps larger than 25 bp were assessed for additional genes. An e value <10^−4^ was used as the threshold for function assignments (17).

The complete Ruchi genome (38,571 bp; 67.7% GC; 3′ overhang GAGTTGCCGGGA) contains 64 predicted ORFs [36 with ascribed function (56%)] and no predicted tRNA genes. Genes 1–24 and 35–64 are encoded on one strand, and genes 25–34 are encoded on the other. Among the predicted genes are four nucleases, endolysin, an immunity repressor (adjacent to tyrosine integrase), and an excise protein as well as RusA-like resolvase. ORFs 14 and 15 are predicted to encode overlapping tail assembly chaperones (111 and 244 aa, respectively) with ORF 14 terminated by a −1 frameshift at nucleotide position 10336. Three predicted ORFs with assigned functions (ORFs 4, 16, 24) and five with unassigned functions (ORFs 1, 21, 22, 39, 47) likely have transmembrane domains. Ruchi shares the highest nucleotide sequence similarity (98.7% identity) with *Arthrobacter* phage Basilisk (Genbank ON260822), which was isolated from Lumpkin County, GA, a year earlier about 20 km from Ruchi’s locale.

## Data Availability

The Ruchi genome can be accessed through NCBI (Genbank OR434022) and sequencing reads can be obtained from the SRA (SRX22366555).
